# Text Book of Abdominal Surgery

**Published:** 1894-12

**Authors:** 


*Text Book of Abdominal Surgery*. By SKENE KEITH, assisted by GEORGE E. KEITH, M.B., C.M. Pp. xiv., 508. Edinburgh: YOUNG J. PENTLAND. 1894.

There are many things that one must deal gently with in the first edition of a book, and amongst these are the inclusion of retro-peritoneal tumours, omental tumours, and mesenteric cysts under "The Peritoneum"; and the somewhat more humorous classification of Cæsarean section, Porro's operation, symphysiotomy, &c., as "Diseases of the Uterus" (*vide* Table of Contents). So too amongst the pardonable errors we may mention the usual mistakes of tympanitis for tympanites (p. 157), and ileum for ilium (pp. 191, 262). Koeberlee frequently appears for Koeberle, and laparo-electrotomy occurs not less than eleven times for laparo-elytrotomy—indeed the latter word is never once put correctly. There is a tendency now and then to repeat what has been said before, and this is most marked on pp. 178 to 180 where we find much that is contained on pp. 167 *et seq*.

We cannot deal so lightly with the questionable taste displayed in many parts of the book, and especially when the authors are inclined to disparage the work of others.

In the preface we are told that the book is "intended to show the present state of our knowledge of abdominal surgery"; but we fear it scarcely does that. Ureteral surgery, which has made great advances of late, scarcely finds a place except as regards the question of stone (mentioned under nephro-lithotomy, p. 276) and accidental division in the removal of an ovarian tumour (p. 365). In the former case (that of stone in the ureter) it is stated (p. 277) that the authors are not aware that stones have been removed from the ureter by a supra-pubic cystotomy; and yet we believe Tuffier has reported two cases. No allusion is made to the cases of Emmet, Berg, Sanger, and others, in which stones have been removed from the ureter through the bladder by dilating the female urethra. Supra-pubic cystotomy too, is not granted a place; and the use of Murphy's buttons, which have practically revolutionised the operation of cholecyst-enterostomy, are not even mentioned.

The book is interspersed with the record of clinical cases which certainly help to exemplify the points which the authors wish to bring out; but in many of these cases it is desirable that references should be given, in order that others may benefit by the perusal of the details of the original report. There are no references throughout the book; and indeed the authors have, as we think, paid too little regard to the acknowledgment of the work of others, for a similarity in phraseology with some books with which we happen to be familiar has struck us, and we readily subscribe to the statement in the preface that the authors are "undoubtedly indebted to others."

The work, as far as it goes, is clearly written; but it is not up to date, and contains little that is original. The best chapters are those on "Tumours of the Ovary" and "Fibroid Tumours of the Uterus," to which about one-fourth of the book is devoted.

No doubt much that we have complained of can be altered in a future edition; but we feel that in its present form it cannot be placed amongst those in the front rank of abdominal surgery.

The publisher has done his part of the work admirably; but although pictorial processes have been lately unduly introduced into some works, especially those of this class, additional plates and diagrams would have been useful in this volume.

